# LMO7-ALK Fusion in a Lung Adenocarcinoma Patient With Crizotinib: A Case Report

**DOI:** 10.3389/fonc.2022.841493

**Published:** 2022-05-19

**Authors:** Yanlong Yang, Hongbo Zheng, Zizhe Li, Shuchen Shi, Lang Zhong, Longlong Gong, Bin Lan

**Affiliations:** ^1^ Department of Cardiothoracic Surgery, Shantou Central Hospital, Shantou, China; ^2^ Medical Department, Genecast Biotechnology Co., Ltd., Wuxi, China

**Keywords:** LMO7-ALK fusion, crizotinib, NSCLC, case report, LUAD

## Abstract

**Background:**

Rearrangements of the anaplastic lymphoma kinase (ALK) gene comprise a small subset of non-small cell lung cancer (NSCLC). Patients with NSCLC harboring ALK fusion proteins are sensitive to ALK tyrosine kinase inhibitors (TKIs). Various fusion partners of ALK are being discovered with the application of next-generation sequencing (NGS).

**Case presentation:**

Here, we report a female patient with metastatic lung adenocarcinoma harboring LMO7-ALK (L15, A20) rearrangement revealed by NGS. The patient received crizotinib as first-line treatment and has achieved partial response with a progression-free survival over 1 year.

**Conclusions:**

We firstly found that the satisfactory response to crizotinib verified the oncogenic activity of LMO7-ALK fusion. Great progression and wide application of NGS facilitate the findings of rare fusion types.

## Background

The identification of driver genes including EGFR mutation and ALK rearrangements greatly promotes the precise therapy for non-small cell lung cancer (NSCLC). With the development of sequencing technology, molecular genotyping has been gradually conducted as a clinical routine to determine the therapeutic regimen. Meanwhile, novel mutations are being identified, providing more treatment opportunities for patients.

ALK is a receptor tyrosine kinase, and its rearrangements occur in about 3%–7% of patients with NSCLC (1). Until now, more than 90 fusion types of ALK have been reported (2), including EML4, KIF5B, KLC1, STRN, TFG, TPM, PPP4R3, GTF2IRD1, VCL, and DCTNA (3–8). The ALK fusion proteins produced by gene arrangements have aberrant kinase activity and promote proliferation and survival of cancer cells through downstream pathways such as RAS-ERK and PI3K-AKT (9). With the access of ALK inhibitors, long-term survival has been realized in patients with advanced NSCLC harboring ALK fusions, with a response rate (RR) of over 80% and a 5-year OS higher than 60% (10, 11).

LMO7-ALK fusion was also reported by previous studies. Ka-Won Noh et al. reported that LMO7-ALK (L15, A20) as a novel ALK fusion was discovered in NSCLC (12). Mengnan Li et al. found that an NSCLC patient with LMO7-ALK (L16, A20) benefited from a second-line ensartinib treatment (13). Here, we report an LMO7-ALK (L15, A20) fusion in a patient with stage IV lung adenocarcinoma who had favorable and durable response to crizotinib treatment.

## Case Presentation

**Figure 1:**
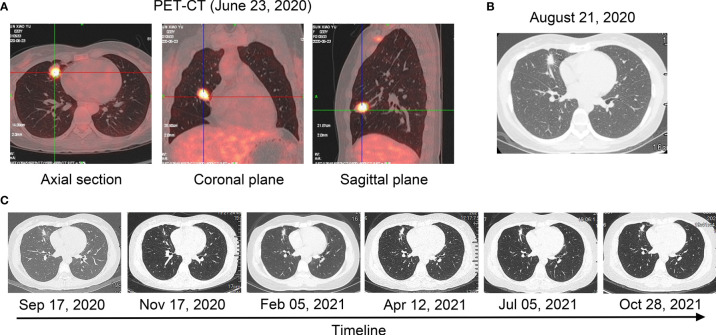
Radiological examinations during the treatment course. **(A)** PET/CT scan before treatment (23 June 2020). **(B)** A computer tomography scan after treatment with crizotinib for 6 weeks (21 August 2020). **(C)** A computer tomography scan after treatment with crizotinib from 17 September 2020 to 28 October 2021.

**Figure 2:**
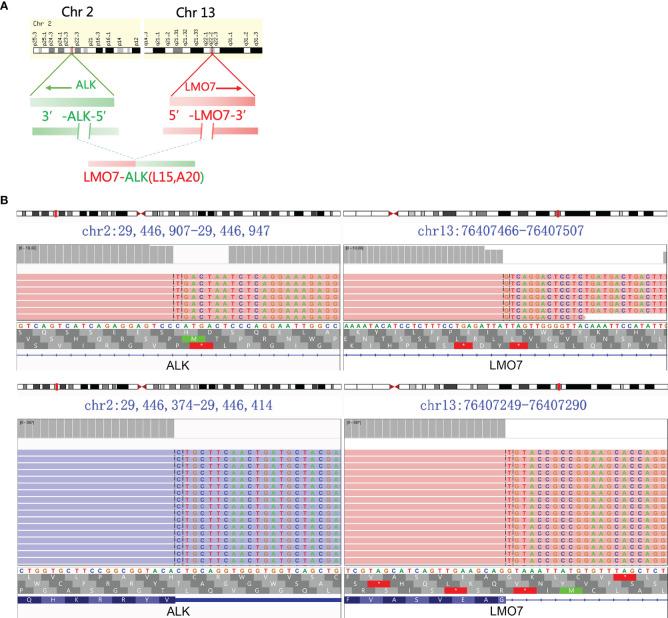
ALK fusion was detected using NGS. **(A)** Schematic representation of LMO7-ALK (L15, A20). **(B)** LMO7-ALK (L15, A20) fusion demonstrated by Integrative Genomics Viewed program.

A 33-year-old woman who had never smoked presented to our hospital with hoarseness in June 2020. There was a family history as her mother died of lung cancer. Positron emission tomography/computed tomography (PET/CT) showed a hypermetabolic mass in the middle lobe of the right lung with metastases of hilar and mediastinal lymph nodes, right supraclavicular lymph nodes, and right ilium (T1cN3M1c, stage IVB, **Figure 1A**). A biopsy through a fiber bronchoscope was performed and pathology revealed adenocarcinoma. Immunohistochemical (IHC) staining indicated positive TTF-1, Napsin A, and CK7, and negative P40 and CK5/6. Formalin-fixed paraffin-embedded (FFPE) tumor samples were assessed by DNA next-generation sequencing (NGS, 122 gene panel, Genecast Biotechnology Co., Ltd.) and RNA NGS (29-gene panel, Genecast Biotechnology Co., Ltd.). The results of DNA and RNA sequencing identified an LMO7-ALK (L15, A20) fusion in the patient. The breakpoints of the two genes were chromosome 2 (29446313–29446354) and LMO7 chromosome 13 (76407282-76407322; **Figures 2A, B**).

The patient started to receive crizotinib (250 mg, twice a day) on 9 July 2020. After 6 weeks, the CT scan demonstrated reduced tumor size and smaller subcarinal lymph nodes (17 × 12 × 12 mm) compared with the previous examination, and evaluation of therapeutic efficiency indicated partial response (PR, −34.2%) (**Figure 1B**). A CT scan on 17 September 2020 showed stable disease (SD, −12%) and smaller subcarinal lymph nodes (12 × 10 × 10 mm) (**Figure 1C**). Follow-up CT scans showed SD (0%) and sizes of subcarinal lymph nodes were almost unchanged until October 2021 (**Figure 1C**). We conducted ctDNA analysis using DNA NGS (122-gene panel, Genecast Biotechnology Co., Ltd.) with peripheral blood in May 2021, while LMO7-ALK rearrangement was not detected. The PFS for the patients has exceeded 1 year.

## Discussion

This is the report of LMO7-ALK (L15, A20) fusion in NSCLC. The LMO7 (LIM-domain only protein 7) gene is located on chromosome 13q22 while ALK is located on chromosome 2p23 (genecard.org). The fusion transcript was generated by the fusion of LMO7 exon 15 and ALK exon 20. In a previous report, LMO7-BRAF (L6, B9) rearrangement was found in papillary thyroid carcinoma. The fusion protein stimulated ERK1/2 phosphorylation and promoted cell growth in a pattern similar to BRAF (14). This demonstrated that LMO7 may be a functional fusion partner for kinases. The cytoplasmic domain of ALK is encoded by exons 17–26, and the most reported breakpoint of ALK is exon 20 (1). All the functional arrangements are the combination of the 5’-end of the fusion partner and 3’-end of ALK, retaining the entire kinase domain of ALK for activity (15). In our case, the breakpoint of ALK is exon 20 without exception, indicating the retention of the entire kinase domain of ALK, and the fusion protein is likely to be generated from inter-chromosomal arrangement similar to TFG-ALK and KIF5B-ALK (1). The fusion protein of LMO7 to ALK is able to drive carcinogenesis and is considered to behave as an oncogenic alteration that displays a favorable response to crizotinib. The repetitive NGS revealed that the LMO7-ALK fusion is cleared in plasma, which was in accordance with the control of disease.

In previous studies, different ALK fusion variants might have different properties. The short forms of EML4-ALK have later stage and poorer prognosis than the long forms (12, 16), while different fusion partners affect drug sensitivity to ALK TKIs (14). The influences of LMO7 on the intrinsic properties of the fusion protein are still unknown. The patient’s exact response to crizotinib and other ALK TKIs, and possible resistance mechanisms need to be followed up.

An increasing number of novel gene fusions are identified with NGS development and application. DNA and RNA NGSs of tumor tissue samples have been widely used (17). In previous gene detection, gene variations of the tumor tissue from the NSCLC patient were assessed using a DNA NGS and the LMO7-ALK fusion was found. To further verify the result, the RNA NGS of the sample was used and the result was consistent with the DNA NGS. With improving DNA sequencing depth and accuracy, ctDNA sequencing is increasingly applied in gene variation detection (18–20), such as gene fusion, single-nucleotide variations, and copy number variation. Owing to tumor tissue disappearance, LMO7-ALK fusion detection using ctDNA sequencing suggested that there was no tumor cell. In the case, application of DNA and RNA NGS of tumor tissues and ctDNA NGS dynamically revealed variation information of the tumor, which provided precise direction for tumor therapy.

There are limitations for this case. We only confirm that the NSCLC case with LMO7-ALK fusion benefitted from crizotinib therapy. The upstream and downstream molecules in this signaling pathway should also be further illustrated.

## Conclusions

In conclusion, LMO7-ALK fusion was verified by the effective treatment of crizotinib in an NSCLC case.

## Data Availability Statement

The original contributions presented in the study are included in the article/**Supplementary Material**. Further inquiries can be directed to the corresponding authors.

## Ethics Statement

Ethical review and approval were not required for the study on human participants in accordance with the local legislation and institutional requirements. The patients/participants provided their written informed consent to participate in this study. Written informed consent was obtained from the individual(s), and minor(s)’ legal guardian/next of kin, for the publication of any potentially identifiable images or data included in this article.

## Author Contributions

YY, HZ, and LZ provided the case and drafted the manuscript. YY and LG analyzed data. SS, LG, and ZL reviewed and amended the manuscript. HZ, LG, and BL amended and designed the manuscript. All authors contributed to the article and approved the submitted version.

## Conflict of Interest

Authors HZ and LG were employed by Genecast Biotechnology Co., Ltd.

The remaining authors declare that the research was conducted in the absence of any commercial or financial relationships that could be construed as a potential conflict of interest.

## Publisher’s Note

All claims expressed in this article are solely those of the authors and do not necessarily represent those of their affiliated organizations, or those of the publisher, the editors and the reviewers. Any product that may be evaluated in this article, or claim that may be made by its manufacturer, is not guaranteed or endorsed by the publisher.
