# Supplementary material for: LMO7-ALK Fusion in a Lung Adenocarcinoma Patient With Crizotinib: A Case Report
Source: Front Oncol. 2022 May 19;12:841493. doi: 10.3389/fonc.2022.841493 (PMC9162556; doi:10.3389/fonc.2022.841493)
Supplement: Supplementary file 1 [file DataSheet_1.docx]

**LMO7-ALK fusion in a lung adenocarcinoma patient with crizotinib: a case report**

**Supplementary material**

**DNA panel sequencing**

FFPE DNA and ctDNA were performed by Genecast Biotechnology Co., Ltd. DNA was sheared with Covaris LE220, and the KAPA Hyper Preparation Kit (Kapa Biosystems, USA) was used to prepare libraries with the fragmented DNA. Targeted region selection was performed with xGen Hybridization and Wash Kit (IDT). The panel was designed to cover 543 genes frequently mutated in common solid tumors in TCGA database. The captured product was sequenced using an Illumina NovaSeq 6000. The DNA panel contains 122 genes: ABCB1, ABCC2, AKT1, AKT2, AKT3, ALK, APC, AR, ARAF, ARID1A, ATIC, ATM, B2M, BRAF, BRCA1, BRCA2, C8orf34, CBR3, CCND1, CCND2, CCND3, CD74, CDA, CDK4, CDK6, CDKN2A, CSF1R, CTNNB1, CYP19A1, CYP2C8, CYP2D6, DDR2, DHFR, DPYD, DYNC2H1, EGFR, EML4, ERBB2(HER2), ERBB4(HER4), ERCC1, ERCC2, ESR1, FBXW7, FGFR1, FGFR2, FGFR3, FLT1(VEGFR1), FLT3, FLT4(VEGFR3), GALNT14, GNA11, GNAQ, GSTP1, HNF4A, HRAS, IDH1, IDH2, JAK1, JAK2, KDR(VEGFR2), KIF5B, KIT, KRAS, MAP2K1(MEK1), MAP2K2(MEK2), MEN1, MET, MLH1, MSH2, MSH6, MTHFR, MTOR, MYC, NF1, NF2, NOTCH1, NR1I3, NRAS, NT5C2, NTRK1, NTRK2, NTRK3, PDGFRA, PDGFRB(PDGFR), PIGF, PIK3CA, PIK3R1, PMS2, POLE, PTCH1, PTEN, RB1, RET, RICTOR, ROS1, RRM1, SDHA, SDHB, SDHC, SDHD, SEMA3C, SLC22A2, SLC31A1, SLC34A2, SLCO1B1, SLCO1B3, SLIT1, SMAD4, SMO, SOD2, STK11(LKB1), TERT, TP53, TPM3, TSC1, TSC2, TYMS, UGT1A1, UMPS, VEGFA, XPC, XRCC1.

**RNA panel sequencing**

FFPE RNA sequencing was performed by Genecast Biotechnology Co., Ltd. RNA was extracted from FFPE samples using the MagPure FFPE RNA/DNA Kit (Magen, China), and FFPE RNA (50-300 ng) was employed for library construction using mRNA-seq Lib Prep Module for Illumina (ABclonal, China). The RNA panel was synthesized by IDT (Integrated DNA Technologies, USA). Hybridization and washing were performed using the xGen Hybridization and Wash Kit and xGen Universal Blockers-TS Mix-96rxn (IDT, USA) according to manufacturer’s instructions. The captured products were sequenced on Illumina NovaSeq 6000. The RNA panel contains 29 genes: ALK, BRAF, CD74, EGFR, EML4, ERG, ESR1, ETV1, ETV4, ETV5, ETV6, EWSR1, FGFR1, FGFR2, FGFR3, FGFR4, KIF5B, MET, NRG1, NTRK1, NTRK2, NTRK3, PDGFRA, PDGFRB, RET, ROS1, SLC34A2, SLC45A3, TPM3.
